# Distinct Distribution of Archaea From Soil to Freshwater to Estuary: Implications of Archaeal Composition and Function in Different Environments

**DOI:** 10.3389/fmicb.2020.576661

**Published:** 2020-10-22

**Authors:** Hualong Wang, Raven Bier, Laura Zgleszewski, Marc Peipoch, Emmanuel Omondi, Atanu Mukherjee, Feng Chen, Chuanlun Zhang, Jinjun Kan

**Affiliations:** ^1^College of Marine Life Sciences, Ocean University of China, Qingdao, China; ^2^Institute of Marine and Environmental Technology, University of Maryland Center for Environmental Science, Baltimore, MD, United States; ^3^Microbiology Division, Stroud Water Research Center, Avondale, PA, United States; ^4^Rodale Institute, Kutztown, PA, United States; ^5^Department of Ocean Science and Engineering, Southern University of Science and Technology, Shenzhen, China; ^6^Shenzhen Key Laboratory of Marine Archaea Geo-Omics, Southern University of Science and Technology, Shenzhen, China; ^7^Southern Marine Science and Engineering Guangdong Laboratory (Guangzhou), Guangzhou, China; ^8^Academy for Advanced Interdisciplinary Studies, Southern University of Science and Technology, Shenzhen, China

**Keywords:** Archaea, composition and distribution, soil, freshwater, estuary, 16S rRNA gene, high-throughput sequencing

## Abstract

In addition to inhabiting extreme territories, Archaea are widely distributed in common environments spanning from terrestrial to aquatic environments. This study investigated and compared archaeal community structures from three different habitats (representing distinct environments): agriculture soils (from farming system trials FST, PA, United States), freshwater biofilms (from White Clay Creek, PA, United States), and estuary water (Chesapeake Bay, United States). High-throughput sequencing of 16S rRNA genes indicated that Thaumarchaeota, Euryarchaeota, Nanoarchaeota, Crenarchaeota, and Diapherotrites were the commonly found dominant phyla across these three environments. Similar to Bacteria, distinct community structure and distribution patterns for Archaea were observed in soils vs. freshwater vs. estuary. However, the abundance, richness, evenness, and diversity of archaeal communities were significantly greater in soils than it was in freshwater and estuarine environments. Indicator species (or amplicon sequence variants, ASVs) were identified from different nitrogen and carbon cycling archaeal groups in soils (Nitrososphaerales, Nitrosotaleales, Nitrosopumilales, Methanomassiliicoccales, Lainarchaeales), freshwater biofilms (Methanobacteria, Nitrososphaerales) and Chesapeake Bay (Marine Group II, Nitrosopumilales), suggesting the habitat-specificity of their biogeochemical contributions to different environments. Distinct functional aspects of Archaea were also confirmed by functional predictions (PICRUSt2 analysis). Further, co-occurrence network analysis indicated that only soil Archaea formed stable modules. Keystone species (ASVs) were identified mainly from Methanomassiliicoccales, Nitrososphaerales, Nitrosopumilales. Overall, these results indicate a strong habitat-dependent distribution of Archaea and their functional partitions within the local environments.

## Introduction

Archaea represent a diverse, abundant and widely distributed group of microorganisms in the biosphere (Karner et al., 2001; Baker et al., 2020). On the basis of cell counts and molecular studies, Archaea account for more than 20% of all prokaryotes in ocean waters (Karner et al., 2001), about 1–5% in surface soil layers (Ochsenreiter et al., 2003; Bates et al., 2011), and probably represent the dominant group of microorganisms in marine subsurface sediments (Lipp et al., 2008). Further, they are abundant in many extreme environments (Klenk et al., 1998; Takai et al., 2008; Kan et al., 2011; Baker et al., 2020). Archaea have a significant impact on biogeochemical cycling (Offre et al., 2013). These microorganisms have evolved a variety of energy metabolisms using organic and/or inorganic electron donors and acceptors (including fixing carbon from inorganic sources) and thus, play crucial roles in global geochemical cycles including influencing greenhouse gas emissions (Offre et al., 2013). For example, methanogenesis and anaerobic methane oxidation are important steps in the carbon cycle that are performed by anaerobic Archaea (Liu and Whitman, 2008; Ferry, 2010; Shen et al., 2019). Although both Archaea and Bacteria contribute to the globally important process of aerobic ammonia oxidation, the wide distribution of ammonia oxidizing Archaea in virtually all investigated aerobic habitats indicates a prominent role for these organisms (Stahl and de la Torre, 2012; Alves et al., 2018). Oxidation of ammonia to nitrite, the first step of nitrification, is performed by aerobic Thaumarchaeota, as well as by some bacterial lineages. Thaumarchaeota is abundant in oceanic plankton and also widely distributed in terrestrial environments (Leininger et al., 2006; Offre et al., 2013). It is becoming apparent that the archaeal communities have much more varied and consequential roles in biogeochemical cycles across different environments than previously thought.

As the overwhelming majority of Archaea resist cultivation in the laboratory, the availability of molecular methods, such as 16S rRNA gene cloning and high-throughput amplicon sequencing, has boosted insight into their astonishing taxonomic and metabolic diversity and omnipresence (Offre et al., 2013; Baker et al., 2020). Different types of non-extremophilic Archaea have been detected in many environments, ranging from terrestrial to marine ecosystems (Auguet et al., 2010; Flemming and Wuertz, 2019). Members of Crenarchaeota and Euryarchaeota are globally distributed, and some lineages, often uncultivated ones, are abundant in waters (Bomberg et al., 2008), soils (Pesaro and Widmer, 2002; Timonen and Bomberg, 2009), and sediments (Schleper et al., 2005; Schleper and Nicol, 2010). For example, two major groups of Euryarchaeota, MG-II and MG-III Archaea are commonly found in estuarine and oceanic waters worldwide (DeLong, 1992; Massana et al., 2000; Xie et al., 2018; Chen et al., 2020). Some MG-II organisms contain rhodopsins which are predicted to use light to boost energy yield or facilitate substrate transport, and are also capable of protein degradation (Iverson et al., 2012; Orsi et al., 2016). Even Bacteria and Archaea perform the same functions, in some habitats Archaea may exhibit greater activity. For example, Herrmann et al. (2008) found that although archaeal and bacterial ammonia monooxygenase genes (*amo*A) had similar relative abundances in freshwater sediment, the enhanced nitrification activity observed in the rhizosphere of aquatic plant (*Littorella uniflora*) was due to ammonia-oxidizing Archaea.

While numerous studies have investigated archaeal distribution and abundance, there is substantial insight to be gained from evaluating the biogeography of this domain with current technology. Most early studies focused on a single environment or with limited spatial scales (Biller et al., 2012; Yao et al., 2013), and sequencing protocols varied among these studies, including the specific primers, sequencing depth, platforms and qualities. For example, Auguet et al. (2010) investigated the global distribution of archaeal communities by using the sequences present in databases at that time which were obtained predominantly by fluorescence in site hybridization (FISH) and denaturant gradient gel electrophoresis (DGGE) band sequencing. Global distribution of specific functional archaeal groups was also researched in earlier studies, including ammonia-oxidizing Archaea (Cao et al., 2013; Alves et al., 2018) and methanogenic Archaea (Wen et al., 2017). However, systematic and detailed investigations on the composition and distribution of whole archaeal communities as well as similarities and differences across different environments (e.g., from soil to fresh water to estuary) by high-throughput sequencing are still lacking. Indeed, little is known about the archaeal composition and distribution in lotic freshwater environments (Bomberg et al., 2008; Auguet et al., 2010) compared to terrestrial (Timonen and Bomberg, 2009; Karimi et al., 2018; Flemming and Wuertz, 2019) and other aquatic environments (e.g., lakes and oceans) (Francis et al., 2005; Offre et al., 2013; Liu et al., 2018; Baker et al., 2020). Filling this knowledge gap would provide important insights into evaluating the biogeography and ecological roles of archaeal communities among distinct ecological environments.

Here we investigated and compared archaeal community structures and their distribution patterns from three different habitats representing distinct environments using identical protocols of high-throughput sequencing analysis. A total of 230 samples were collected in this study: 95 agriculture soil samples from farming system trials (FST) at Rodale Institute, Pennsylvania, United States, 59 freshwater biofilms from White Clay Creek, Pennsylvania, United States, and 76 surface water samples from Chesapeake Bay, the biggest estuary in the North America. Deep sequencing showed more than 4,000 unique archaeal 16S rRNA gene sequences, unveiled dominant archaeal taxa, and also indicated distinct species distribution across these environments. The archaeal abundance, richness, evenness, and diversity were compared among these environments. Higher archaeal abundance and diversities were associated with soils than freshwater biofilms and estuarine environments. In addition, indicator species, co-occurrence networks, and potential ecological functions of archaeal groups from distinct environments were also explored and discussed.

## Materials and Methods

### Sample Collection

**FIGURE 1:**
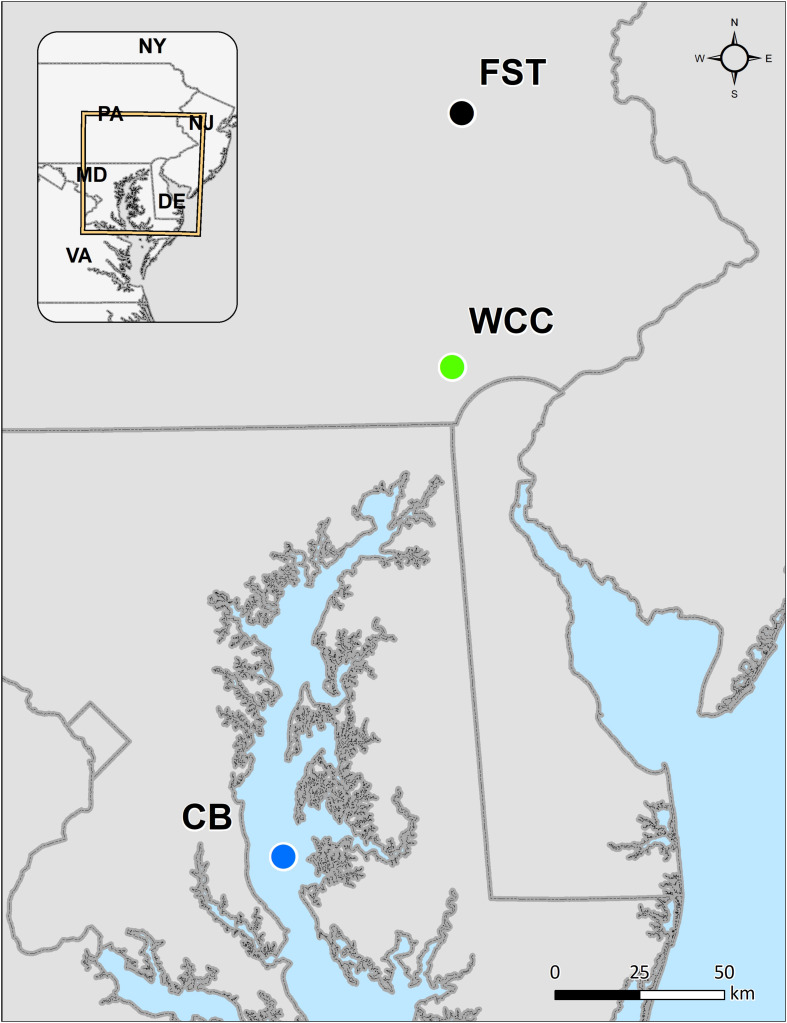
Map of our sampling from agricultural soils (farming system trials, FST), freshwater biofilms at White Clay Creek (WCC), and the Chesapeake Bay estuary (CB).

In total, 230 samples were collected from three habitats representing terrestrial, lotic and estuarine environments: agriculture soils (95 samples), freshwater biofilms (59 samples), and estuary water (76 samples) (Figure 1). Agricultural soils were collected from Rodale Institute’s Farming Systems Trial (labeled as FST in this study) in Kutztown, Pennsylvania, United States (40.5509N, −75.7297W) that spans conventional and organic agriculture (Figure 1). Soil samples were collected in January 2019 using a 4.5 cm soil probe and taken to 1 m depth. Each soil core was sectioned into the following depth intervals: 0–10, 10–20, 20–30, and 30–60 cm. For each sample, four different soil cores spanning one agricultural treatment field were homogenized. A subsample was stored at −80°C until DNA extraction. In freshwaters especially upstream lotic environments, biofilms are the primary and dominant life forms of microorganisms, and therefore freshwater biofilms were collected from a flume study using continuous inputs of surface water from White Clay Creek (39.8592N, −75.7837W, southeastern Pennsylvania, United States; labeled as WCC) in summer 2018 (Figure 1). Biofilms were grown on autoclaved rocks in the flumes and collected on days 2, 5, 9, 13, and 20 of their development during July and August. Four rocks were swabbed on 37 cm^2^ of surface area and frozen at −80°C until DNA extraction. The surface water samples in Chesapeake Bay (labeled as CB) were collected at seven stations along the middle axis of the Bay in February/March, May/June, August, and October from 2003 to 2005 (Figure 1). Details of estuary water sample collection, sampling locations and sample preparation have been described previously (Kan, 2006). In brief, 500 ml surface water (below 2 m) were taken at each sampling station and filtered immediately through 0.2 μm Millipore polycarbonate filters (47 mm diameter; Millipore Corporation, Billerica, MA, United States). The filters were stored at −80°C. Environmental parameters were summarized to describe the three habitats (Supplementary Table S1), although different environmental variables were measured in each habitat and data collection was performed differently. The average measurements values of the variables for agricultural soils, freshwater biofilms, and estuary waters were grouped according to different sampling depth, time points, and sites. Although the samples from these 3 different habitats were not collected at the same time, we believe they were good representatives for each environment based on the sample collection by including different farming practices and depth for FST (Rodale Institute’s Farming Systems Trial, labeled as FST in this study), the time series of WCC biofilm development, and water samples across space and time in the Chesapeake Bay. However, it is also important to note that the freshwater biofilm and soil environments represent distinct seasons while estuary samples span seasons. Thus, we interpret community differences across environments as specific to the season or seasons when collected.

### DNA Extraction and High-Throughput Sequencing

Environmental DNA from soils (FST) and biofilms (WCC) were extracted using DNeasy PowerSoil kits (Qiagen, Hilden, Germany) following the manufacturer’s instructions. DNA extraction from surface water samples (CB) followed the protocol described previously (Kan et al., 2006). DNA quantity was assessed using a NanoDrop 2000 spectrophotometer (Thermo Fisher Scientific Inc., Waltham, MA). Library preparation and sequencing followed the 16S Metagenomic Sequencing Library Preparation protocol from Illumina^1^. Hypervariable region 4 (V4) of the SSU rRNA gene primers are now widely employed for defining microbial diversity (including both Archaea and Bacteria) across many different environments by high-throughput sequencing (Caporaso et al., 2011), including the Earth Microbiome Project’s exploration of the global microbiome (Gilbert et al., 2014). The V4 variable region of the 16S rRNA genes was amplified using the universal forward primer 515f (5′-GTGYCAGCMGCCGCGGTAA-3′) (Parada et al., 2016) and universal reverse primer 806r (5′-GGACTACNVGGGTWTCTAAT-3′) (Apprill et al., 2015). PCR reactions contained 25 μl 2x Premix Taq, 1 μl each primer (10 mM) and 3 μl environmental DNA (20 ng/μl) template in a volume of 50 μl, and were amplified with following thermocycling program: 5 min at 94°C for initialization; 30 cycles of 30 s denaturation at 94°C, 30 s annealing at 52°C, and 30 s extension at 72°C; followed by 10 min final elongation at 72°C. Sequencing libraries were generated by using NEBNext Ultra II DNA Library Prep Kit for Illumina (New England Biolabs, MA, United States) following manufacturer’s recommendations. Each library was quantified using Qubit 2.0 fluorometer double-stranded DNA high sensitivity DNA kit (Thermo Fisher Scientific, MA, United States). Then libraries were normalized and mixed in equidensity ratios. High-throughput sequencing of 16S rRNA genes was performed at Magigene (Magigene Biotechnology Co. Ltd., Guangzhou, China) on an Illumina Nova6000 platform (paired-end 250 bp mode), following the manufacturer’s guidelines. Raw sequencing data obtained in this study are available through the GenBank database under the accession numbers: PRJNA635685 (for FST soil samples), PRJNA631093 (for WCC biofilm samples), and PRJNA576689 (for CB).

### Sequence Analyses

The QIIME 2 software package (version 2019.10) was used to process the raw sequence data (Bolyen et al., 2019). In brief, a total of 55,780,358 reads were obtained from these 230 samples after demultiplexing. Primers were removed with q2-DADA2 (Callahan et al., 2016), and the reads were trimmed to the same length (forward at 180 bp and reverse at 200 bp). q2-DADA2 was also used for denoising, filtering, merging, and chimera removal from these sequences and generate amplicon sequence variants (ASVs). The statistics of quality control filtering of 16S rRNA gene sequences in our samples were showed in Supplementary Table S2. A Naïve Bayes classifier artifact^2^ was applied to assign the ASVs to taxa at 99% using the Silva classifier 132-99-515-806 dataset (Quast et al., 2012). For all ASV-based analyses, the original ASV table was rarified to a depth of 100,000 sequences per sample in order to minimize the sampling effects. An alpha rarefaction analysis at a sampling depth of 100,000 sequences was analyzed. The rarefaction curves clearly showed that our samples were sequenced to a sufficient depth in regard to prokaryotic diversity from soil to estuary (Supplementary Figure S1). In total, we obtained 4065 ASVs which were affiliated with the archaeal sequences and these were further evaluated. The QIIME 2 package was also used to generate Bray-Curtis distance matrices and α-diversity metrics including evenness, observed ASVs, and Shannon-Wiener diversity.

### Phylogenetic Analyses

Phylogenetic analysis was implemented in the Molecular Evolutionary Genetics Analysis (MEGA) X software (Kumar et al., 2018). Multiple sequence alignment was carried out using CLUSTAL W function (Thompson et al., 1994) with default parameters, and phylogenetic trees of 16S rRNA genes were reconstructed using the neighbor-joining method (Saitou and Nei, 1987). Bootstrap values were calculated with 1,000 re-sampling. The 16S rRNA gene sequence of *Escherichia coli* (NR 024570.1) was used as out-group for the analysis.

### Co-occurrence Network Analysis

Relative abundance of archaeal ASVs were used to construct a co-occurrence network for each dataset from 3 environments. To avoid potentially erroneous sequences and improve interpretability of the dataset, we filtered out ASVs that were presented in fewer than three samples, and whose summed relative abundance was less than 0.1% in each specific network inference. All network constructions were done in R (R Core Team, 2020) (version 3.6.1) using the package “fdrtool” and “igraph” (Williams et al., 2014). We adapted the network construction code at GitHub^3^. The false discovery rate was estimated and corrected by the package “fdrtool.” Co-occurrence networks for each environment were constructed using only statistically significant (*P* < 0.01) and robust (Spearman’s correlation coefficient > | 0.6|) correlations (Barberán et al., 2012). Network visualization and topological analysis were carried out in Gephi (version 0.9.2) (Bastian et al., 2009). Other information regarding nodes (archaeal taxa), including taxonomy and relative abundances, were also imported into Gephi.

### Indicator Taxa Analysis

Indicator taxa were identified for each habitat based on their specificity and fidelity to the environment. This analysis was conducted using the “multipatt” function in R-package “indicspecies” (Cáceres and Legendre, 2009) with 999 permutations and function “r.g” to account for unequal groups through correction of Pearson’s phi coefficient of association. ASVs were selected as good indicators of a particular environment if the indicator value statistic was > 0.3 and *P* < 0.05 as previously recommended (Dufrêne and Legendre, 1997).

### Prediction of Functional Content From Archaeal Communities

The potential functions of archaeal communities were inferred from the 16S rRNA gene high-throughput sequencing data by using PICRUSt2 (phylogenetic Investigation of Communities by Reconstruction of Unobserved States) (Douglas et al., 2019). We predicted KEGG orthology (KO) metagenomes, enzyme commission (EC) metagenomes and MetaCyc pathway abundances through a QIIME 2 module called q2-picrust2^4^. PICRUSt2 uses the 16S rRNA marker gene data to query a reference database for the closest reference genome available. Genomic-driven inference of function is then used to predict gene families, which are combined to estimate the composite metagenome. Briefly, a PICRUSt2-compatible ASV table was constructed in QIIME2. The accuracy for the predicted metagenome was tested through the Nearest Sequenced Taxon Index (NSTI), reflecting the presence of reference genomes that are closely related to the samples in the analysis.

### Statistical Analyses

Statistical analyses were completed with R statistical software (version 3.6.1). Differences between major archaeal groups (phylum level) were compared using a one-way ANOVA (*P* = 0.01). Tukey’s *post hoc* tests were used to test statistical significance (*P* ≤ 0.01) of pair-wise comparisons. Calculation of alpha diversity (including Shannon-Wiener diversity, richness and evenness) of archaeal communities was done using the “diversity” function in the “vegan” package (Oksanen et al., 2019). Non-metric multidimensional scaling (NMDS) was used to assess differences across the three environments in community structures (Bacteria and Archaea, Bacteria only, and Archaea only). Differences of archaeal communities from three different habitats were further tested by analysis of similarities (ANOSIM). Both NMDS and ANOSIM were performed using the “metaMDS” and “anosim” functions in the “vegan” R package, respectively.

## Results

### Uneven Diversity of Archaeal Community Across Three Habitats

**FIGURE 2:**
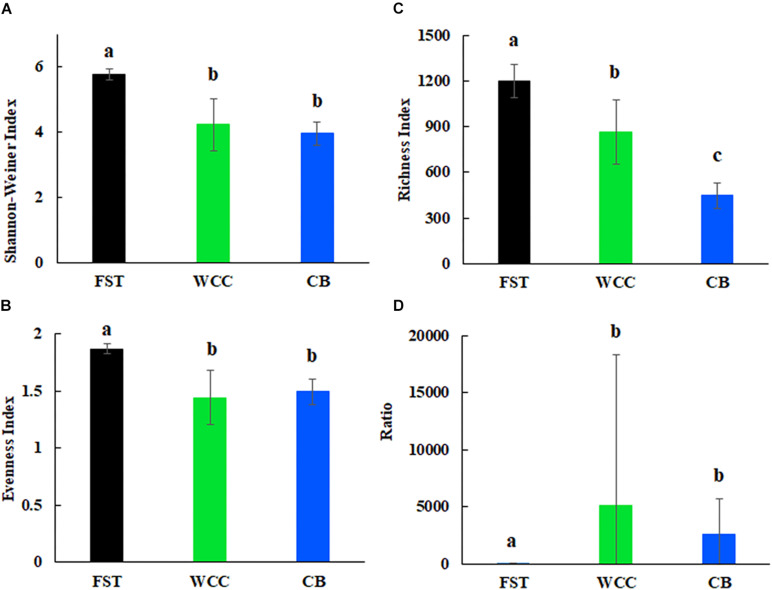
Alpha diversity for agricultural soils (FST, *n* = 95), freshwater biofilms (WCC, *n* = 59) and estuary (CB, *n* = 76): **(A)** Shannon-Weiner index; **(B)** evenness; **(C)** observed richness; and **(D)** ratio of relative abundance of bacteria to archaea. Bars represent mean with standard errors. Different letters above the bars indicate significant differences between habitats (*P* < 0.05), whilst shared letters indicate no significant difference.

Archaea comprised a broad diversity of taxa that were clearly uneven in their distribution across agriculture soils, freshwater biofilms, and estuary waters (Figure 2). Generally, alpha diversity of archaeal communities (Shannon-Wiener, ASV observed richness and evenness indices) were distinct among habitats (Kruskal-Wallis, *P* < 0.05) (Figure 2). Shannon-Wiener diversity of archaeal community was significantly greater in samples collected in agriculture soils than those collected at freshwater biofilms and estuarine surface waters (*P* < 0.05; Figure 2A). Similarly, evenness of archaeal communities was significantly higher in agriculture soils than in freshwater biofilms and estuarine surface water (*P* < 0.05; Figure 2B). Observed ASV richness in agriculture soils was also significantly higher than those in freshwater biofilms and estuary water samples (*P* < 0.05; Figure 2C). In addition, ASV richness in freshwater biofilms was significantly higher than that in estuarine surface waters (*P* < 0.05; Figure 2C). The ratio of total relative abundance of Bacteria to Archaea varied significantly for each habitat (*P* < 0.05; Figure 2D). The Bacteria:Archaea ratio in the agriculture soil samples was significantly lower compared to samples collected from the freshwater biofilms and estuary water, while there was no distinct difference between the freshwater biofilm samples and the estuarine surface water samples (Figure 2D). The low ratio of Bacteria:Archaea showed that Archaea was more abundant in the soil environment compared to the freshwater biofilms and estuary water.

### Detailed Archaeal Community Structure

**FIGURE 3:**
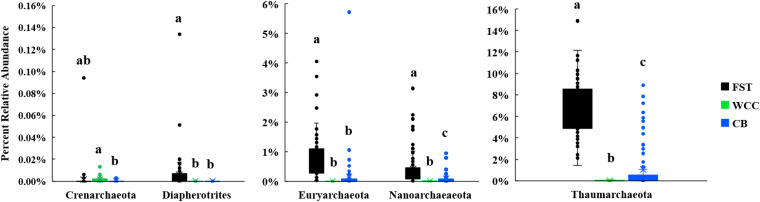
Boxplots for major archaeal phyla from three environments [agricultural soils (FST, *n* = 95), freshwater biofilms (WCC, *n* = 59) and estuary (CB, *n* = 76)]. Different letters above the bars indicate significant differences between habitats (*P* < 0.05), whilst shared letters indicate no significant difference.

The percentage of total 16S rRNA gene sequences dominated by Archaea varied in the three habitats: soils (2.03–17.10%), freshwater biofilms (<0.01–0.16%), and estuary water (0.01–9.39%) (Supplementary Table S2). The archaeal communities were dominated by Thaumarchaeota, Euryarchaeota, Nanoarchaeota, Crenarchaeota, and Diapherotrites (Figure 3). In addition, the relative abundances of these major archaeal groups varied among habitats (Figure 3). Euryarchaeota, Thaumarchaeota and Diapherotrites were more abundant in agriculture soils than in the freshwater biofilms and estuary water (*P* < 0.01), while Crenarchaeota were more predominant in freshwater biofilms compared to estuary water (*P* < 0.01) (Figure 3). The proportion of Thaumarchaeota and Nanoarchaeota was significantly different across the three environments and exhibited the same distribution: they were more abundant in soils, than in estuary water, and were even less abundant in freshwater biofilms (*P* < 0.01) (Figure 3). In total, archaeal communities had the highest relative abundance in soils compared to the other two environments, and the major group was Thaumarchaeota (Figure 3).

There was a clear phylogenetic shift of major groups of archaeal communities across three habitats (Supplementary Table S3 and Supplementary Figures S2A–E). For example, within the phylum Thaumarchaeota, Nitrosopumilaceae (ASV3197) dominated the archaeal communities in the Chesapeake Bay, but was essentially absent, or nearly so, in freshwater biofilms and agricultural soil environments. This implies that the organism represented by phylotype ASV3197 is probably a typical estuarine/brackish species. Two other members of Nitrosopumilaceae (ASV54 and ASV64) were dominant in the freshwater biofilms, yet they were nearly undetected in estuary waters. Furthermore, ASV66 (Nitrosotaleaceae) and ASV64 and 57 (Nitrosopumilaceae) were dominant in the soil environments (Supplementary Table S3 and Supplementary Figure S2E). Similar results were also observed in the phylum Euryarchaeota (Supplementary Figure S2C). Two members of Marine Group II (ASV15 and ASV3190) dominated the Euryarchaeota groups in the estuary waters, while one member of Methanobacteriaceae (ASV3189) dominated in the freshwater biofilms. In soils, there were different dominant taxa within Euryarchaeota groups, including 4 members of Thermoplasmata (ASV16, ASV17, ASV19, and ASV21) (Supplementary Table S3).

### Distribution Patterns of Archaeal Communities

**FIGURE 4:**
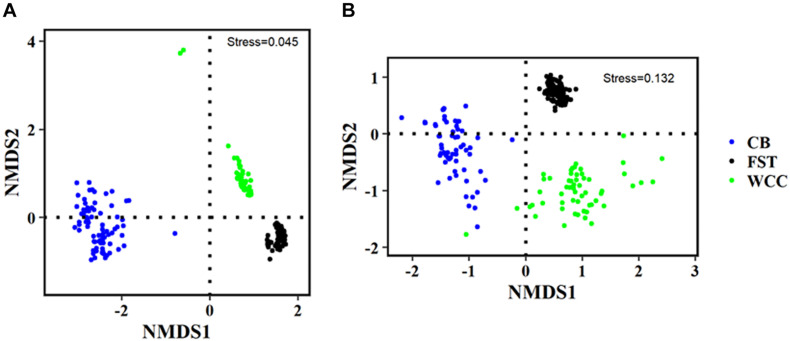
Non-metric multidimensional scaling (NMDS) plots of different communities. **(A)** Bacteria only; and **(B)** Archaea only. FST, agricultural soils; WCC, freshwater biofilms; CB, estuary.

Archaeal communities from agriculture soils, freshwater biofilms, and estuary water showed clear visual separation from each other in regard to the distribution patterns of the microbial community compositions (NMDS, Figure 4 and Supplementary Figure S3). The three different habitats were characterized based on their physico-chemical properties (Supplementary Table S1), and habitat classification was a strong structuring factor of the microbial assemblages and communities clearly grouped according to their environmental types (ANOSIM test, *P* < 0.01) (Figure 4). The partitioned distribution patterns were observed for bacterial communities in soils vs. freshwater vs. estuary (Figure 4A). Similar to Bacteria, global variation of archaeal communities was strongly separated based on habitats: archaeal communities showed distinct distribution patterns across soils, freshwater, and estuary (Figure 4B). The abundance-weighted percentage of Archaea used for NMDS analysis accounts for a low percentage (3.75%) of combined Bacteria and Archaea. Therefore, NMDS plots of Bacteria only and combined Archaea and Bacteria were quite similar to each other with almost the same stress index (Figure 4A, 0.0450; Supplementary Figure S3, 0.0449). The ANOSIM statistic results further confirmed a clear separation of archaeal communities across 3 sampling environments (*R* = 0.9134, *P* = 0.0001).

### Archaeal Networks

**FIGURE 5:**
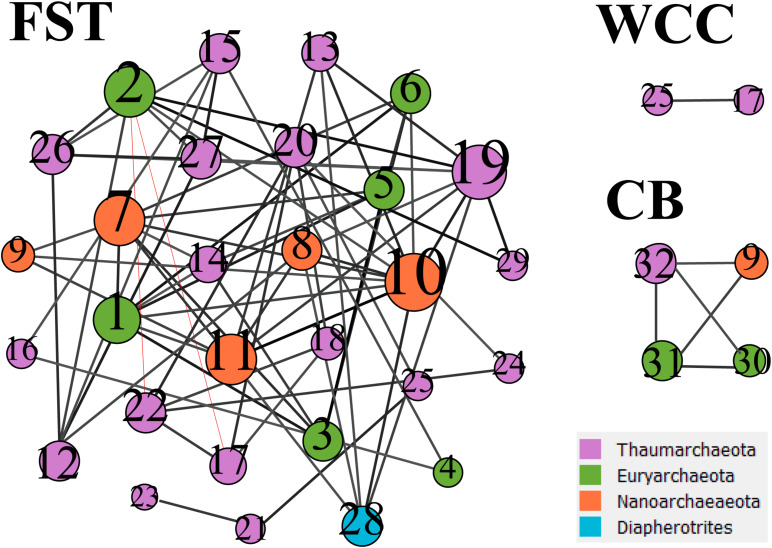
Co-occurrence networks from three environments. FST, agricultural soils; WCC, freshwater biofilms; CB, estuary. The networks were based on archaeal ASVs which have occurred at least in three samples from each environment. Node size represents the number of associations between nodes. The number in the nodes refers to the taxa listed in Table 1. Black lines between nodes represent positive correlations, and red lines represent negative correlations.

Co-occurrence network analysis showed that archaeal associations were distinct in three habitats. Stronger significant relationships among archaeal communities were observed in the agriculture soils (29 taxa with 68 correlations) compared to the freshwater biofilms (2 taxa with 1 correlation) and estuary surface waters (4 taxa with 5 correlations) (Figure 5). Those key archaeal taxa with the highest number of associations in soils were affiliated with Woesearchaeia (Nanoarchaeota; 4 members), Nitrososphaeraceae (Thaumarchaeota) and Methanomassiliicoccales (Euryarchaeota, 5 members) (Table 1). Four taxa that occurred in the archaeal networks from estuary waters belonged to Marine Group II (Euryarchaeota) and Nitrososphaeria and Woesearchaeia (Nanoarchaeota), while two members of freshwater networks were both affiliated to the Nitrososphaeraceae (Thaumarchaeota) (Table 1). Our results clearly showed that habitat differences could significantly influence the archaeal interactions and networks across different environments.

**TABLE 1:** Key species/gatekeepers for three habitats (Archaea only).

Environments	Labels in network	id	Phylum	Class	Order	Family	Genus	Number of samples it was occurred	Total reads in each habitat	Degree
FST	10	ASV41	Nanoarchaeaeota	Woesearchaeia	Uncultured euryarchaeote	Uncultured euryarchaeote	Uncultured euryarchaeote	93	19,274	10
	19	ASV57	Thaumarchaeota	Nitrososphaeria	Nitro- sosphaerales	Nitro- sosphaeraceae	Candidatus Nitrososphaera	95	90,605	9
	2	ASV16	Euryarchaeota	Thermoplasmata	Methanomassi- liicoccales	Uncultured	Uncultured archaeon	65	23,254	8
	7	ASV32	Nanoarchaeaeota	Woesearchaeia	Candidatus Staskawiczbacteria bacterium	Candidatus Staskawiczbacteria bacterium	RIFOXYA2 _FULL_32_7	93	8,360	8
	11	ASV42	Nanoarchaeaeota	Woesearchaeia	NA	NA	NA	88	7,101	8
	1	ASV12	Euryarchaeota	Thermoplasmata	Marine Group II	Uncultured archaeon	Uncultured archaeon	83	2,947	7
	3	ASV17	Euryarchaeota	Thermoplasmata	Methanomassi- liicoccales	NA	NA	95	12,145	5
	5	ASV19	Euryarchaeota	Thermoplasmata	Uncultured	Uncultured archaeon	Uncultured archaeon	95	17,243	5
	6	ASV21	Euryarchaeota	Thermoplasmata	NA	NA	NA	95	23,126	5
	8	ASV34	Nanoarchaeaeota	Woesearchaeia	Nanoarchaeota archaeon SCGC AAA011-D5	Nanoarchaeota archaeon SCGC AAA011-D5	Nanoarchaeota archaeon SCGC AAA011-D5	47	573	5
WCC	17	ASV54	Thaumarchaeota	Nitrososphaeria	Nitro- sosphaerales	Nitro- sosphaeraceae	Candidatus Nitrocosmicus	45	899	1
	25	ASV64	Thaumarchaeota	Nitrososphaeria	Nitro- sosphaerales	Nitro- sosphaeraceae	NA	30	655	1
CB	31	ASV3190	Euryarchaeota	Thermoplasmata	Marine Group II	Marine Group II_ unidentified	Marine Group II_unidentified	45	2,713	3
	32	ASV3197	Thaumarchaeota	Nitrososphaeria	Nitrosopumilales	Nitrosopumilaceae	Candidatus Nitrosopumilus	4	72	3
	30	ASV15	Euryarchaeota	Thermoplasmata	Marine Group II	Marine Group II_ unidentified	Marine Group II_unidentified	75	77,835	2
	9	ASV40	Nanoarchaeaeota	Woesearchaeia	Woesearchaeia_ unidentified	Woesearchaeia_ unidentified	Woesearchaeia_ unidentified	12	291	2

*FST = agricultural soils, WCC = freshwater biofilms, CB = estuary.*

### Archaeal Indicator ASVs

**FIGURE 6:**
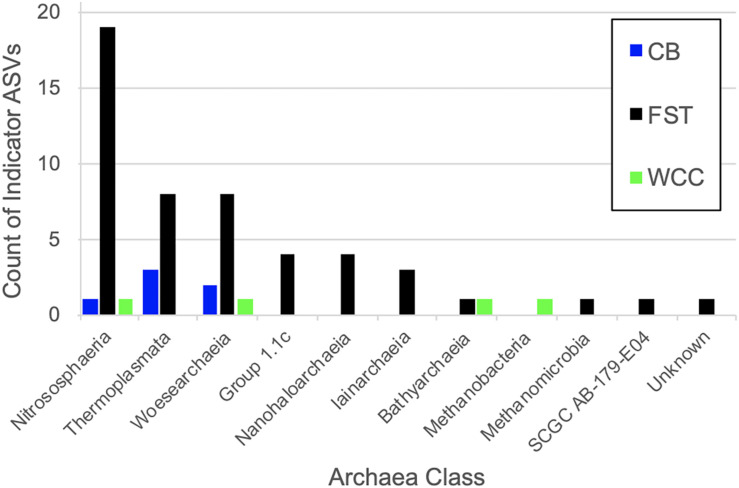
Classes of archaeal ASVs identified as indicators for each environment type. FST, agricultural soils; WCC, freshwater biofilms; CB, estuary.

Archaea ASVs that were indicators for each habitat type represented five phyla and ten classes, with the majority of ASV indicators belonging to uncultured or unidentified species (Table 2 and Figure 6). The number of indicator ASVs for each habitat varied widely with agricultural soils having 50 indicators while freshwater biofilms and estuaries had four and six indicator ASVs, respectively. Nitrososphaeria was the class containing the most ASVs indicative of agricultural soils (19 indicators), followed by classes Thermoplasmata and Woesearchaeia each with eight indicator ASVs. Indicator ASVs for estuaries were from classes Thermoplasmata (3 indicators), Woesearchaeia (2 indicators), and Nitrososphaeria (1 indicator). Freshwater biofilms had one archaeal indictor each from the classes Nitrososphaeria, Woesearchaeia, Bathyarchaeia, and Methanobacteria. Methanobacteria was the only class with an indicator ASV that was restricted to aquatic environments, occurring only for freshwater biofilms. Agricultural soils also contained a methanogenic ASV indicator which was of class Methanomicrobia.

**TABLE 2:** Archaeal indicator ASVs for three sampling habitats.

Habitats	id	Phylum	Class	Order	Family	Genus	Species	Indicator value index	*P*
FST	ASV2	Crenarchaeota	Bathyarchaeia	NA	NA	NA	NA	0.153	0.04
FST	ASV4	Diapherotrites	Iainarchaeia	Iainarchaeales	Diapherotrites archaeon SCGC AAA011-K09	Diapherotrites archaeon SCGC AAA011-K09	Diapherotrites archaeon SCGC AAA011-K09	0.304	0.001
FST	ASV5	Diapherotrites	Iainarchaeia	Iainarchaeales	Diapherotrites archaeon SCGC AAA011-N19	Diapherotrites archaeon SCGC AAA011-N19	Diapherotrites archaeon SCGC AAA011-N19	0.379	0.001
FST	ASV7	Diapherotrites	Iainarchaeia	Iainarchaeales	Uncultured archaeon	Uncultured archaeon	Uncultured archaeon	0.236	0.001
FST	ASV10	Euryarchaeota	Methanomicrobia	Methanosarcinales	Methanosarcinaceae	Methanosarcina	NA	0.197	0.014
FST	ASV12	Euryarchaeota	Thermoplasmata	Marine Group II	Uncultured archaeon	Uncultured archaeon	Uncultured archaeon	0.616	0.001
FST	ASV13	Euryarchaeota	Thermoplasmata	Marine Group II	Uncultured euryarchaeote	Uncultured euryarchaeote	Uncultured euryarchaeote	0.279	0.001
FST	ASV14	Euryarchaeota	Thermoplasmata	Marine Group II	Uncultured haloarchaeon	Uncultured haloarchaeon	Uncultured haloarchaeon	0.314	0.001
FST	ASV17	Euryarchaeota	Thermoplasmata	Methanomassi- liicoccales	NA	NA	NA	0.673	0.001
FST	ASV16	Euryarchaeota	Thermoplasmata	Methanomassi- liicoccales	Uncultured	Uncultured archaeon	Uncultured archaeon	0.327	0.001
FST	ASV21	Euryarchaeota	Thermoplasmata	NA	NA	NA	NA	0.68	0.001
FST	ASV18	Euryarchaeota	Thermoplasmata	Uncultured	Crenarchaeote SRI-298	Crenarchaeote SRI-298	Crenarchaeote SRI-298	0.239	0.001
FST	ASV19	Euryarchaeota	Thermoplasmata	Uncultured	Uncultured archaeon	Uncultured archaeon	Uncultured archaeon	0.683	0.001
FST	ASV22	Nanoarchaeaeota	Nanohaloarchaeia	Aenigmarchaeales	Uncultured archaeon	Uncultured archaeon	Uncultured archaeon	0.36	0.001
FST	ASV25	Nanoarchaeaeota	Nanohaloarchaeia	Deep sea euryarchaeotic group (DSEG)	NA	NA	NA	0.153	0.035
FST	ASV24	Nanoarchaeaeota	Nanohaloarchaeia	Deep sea euryarchaeotic group (DSEG)	Uncultured archaeon	Uncultured archaeon	Uncultured archaeon	0.197	0.012
FST	ASV26	Nanoarchaeaeota	Nanohaloarchaeia	NA	NA	NA	NA	0.4	0.001
FST	ASV35	Nanoarchaeaeota	Woesearchaeia	Archaeon GW2011 _AR13	Archaeon GW2011_AR13	Archaeon GW2011_AR13	Archaeon GW2011 _AR13	0.292	0.001
FST	ASV28	Nanoarchaeaeota	Woesearchaeia	Candidatus Diapherotrites archaeon ADurb.Bin253	Candidatus Diapherotrites archaeon ADurb.Bin253	Candidatus Diapherotrites archaeon ADurb.Bin253	Candidatus Diapherotrites archaeon ADu	0.36	0.001
FST	ASV29	Nanoarchaeaeota	Woesearchaeia	Candidatus Nomurabacteria bacterium RIFCSPLOWO2 _02_FULL _42_17	Candidatus Nomurabacteria bacterium RIFCSPLOWO2 _02_FULL_42_17	Candidatus Nomurabacteria bacterium RIFCSPLOWO2 _02_FULL _42_17	D	0.248	0.001
FST	ASV32	Nanoarchaeaeota	Woesearchaeia	Candidatus Staskawiczbacteria bacterium RIFOXYA2_FULL _32_7	Candidatus Staskawiczbacteria bacterium RIFOXYA2_FULL _32_7	Candidatus Staskawiczbacteria bacterium RIFOXYA2_FULL _32_7	Candi	0.514	0.001
FST	ASV42	Nanoarchaeaeota	Woesearchaeia	NA	NA	NA	NA	0.341	0.001
FST	ASV34	Nanoarchaeaeota	Woesearchaeia	Nanoarchaeota archaeon SCGC AAA011-D5	Nanoarchaeota archaeon SCGC AAA011-D5	Nanoarchaeota archaeon SCGC AAA011-D5	Nanoarchaeota archaeon SCGC AAA011-D5	0.381	0.001
FST	ASV38	Nanoarchaeaeota	Woesearchaeia	Uncultured archaeon CLEAR-15	Uncultured archaeon CLEAR-15	Uncultured archaeon CLEAR-15	Uncultured archaeon CLEAR-15	0.226	0.002
FST	ASV41	Nanoarchaeaeota	Woesearchaeia	Uncultured euryarchaeote	Uncultured euryarchaeote	Uncultured euryarchaeote	Uncultured euryarchaeote	0.408	0.001
FST	ASV46	Thaumarchaeota	Group 1.1c	NA	NA	NA	NA	0.389	0.001
FST	ASV43	Thaumarchaeota	Group 1.1c	Uncultured archaeon	Uncultured archaeon	Uncultured archaeon	Uncultured archaeon	0.228	0.001
FST	ASV44	Thaumarchaeota	Group 1.1c	Uncultured crenarchaeote	Uncultured crenarchaeote	Uncultured crenarchaeote	Uncultured crenarchaeote	0.341	0.001
FST	ASV45	Thaumarchaeota	Group 1.1c	Uncultured thaumarchaeote	Uncultured thaumarchaeote	Uncultured thaumarchaeote	Uncultured thaumarchaeote	0.287	0.001
FST	ASV72	Thaumarchaeota	NA	NA	NA	NA	NA	0.445	0.001
FST	ASV48	Thaumarchaeota	Nitrososphaeria	Nitrosopumilales	Nitrosopumilaceae	Candidatus Nitrosoarchaeum	Uncultured archaeon	0.236	0.001
FST	ASV49	Thaumarchaeota	Nitrososphaeria	Nitrosopumilales	Nitrosopumilaceae	Candidatus Nitrosotenuis	NA	0.535	0.001
FST	ASV50	Thaumarchaeota	Nitrososphaeria	Nitrosopumilales	Nitrosopumilaceae	Uncultured archaeon	Uncultured archaeon	0.243	0.001
FST	ASV54	Thaumarchaeota	Nitrososphaeria	Nitrososphaerales	Nitrososphaeraceae	Candidatus Nitrocosmicus	NA	0.602	0.001
FST	ASV53	Thaumarchaeota	Nitrososphaeria	Nitrososphaerales	Nitrososphaeraceae	Candidatus Nitrocosmicus	Uncultured bacterium	0.216	0.002
FST	ASV57	Thaumarchaeota	Nitrososphaeria	Nitrososphaerales	Nitrososphaeraceae	Candidatus Nitrososphaera	NA	0.559	0.001
FST	ASV55	Thaumarchaeota	Nitrososphaeria	Nitrososphaerales	Nitrososphaeraceae	Candidatus Nitrososphaera	Uncultured crenarchaeote	0.659	0.001
FST	ASV56	Thaumarchaeota	Nitrososphaeria	Nitrososphaerales	Nitrososphaeraceae	Candidatus Nitrososphaera	Unidentified archaeon	0.371	0.001
FST	ASV58	Thaumarchaeota	Nitrososphaeria	Nitrososphaerales	Nitrososphaeraceae	Metagenome	Metagenome	0.649	0.001
FST	ASV64	Thaumarchaeota	Nitrososphaeria	Nitrososphaerales	Nitrososphaeraceae	NA	NA	0.877	0.001
FST	ASV59	Thaumarchaeota	Nitrososphaeria	Nitrososphaerales	Nitrososphaeraceae	Uncultured bacterium	Uncultured bacterium	0.809	0.001
FST	ASV60	Thaumarchaeota	Nitrososphaeria	Nitrososphaerales	Nitrososphaeraceae	Uncultured compost archaeon	Uncultured compost archaeon	0.581	0.001
FST	ASV61	Thaumarchaeota	Nitrososphaeria	Nitrososphaerales	Nitrososphaeraceae	Unidentified archaeon SCA1150	Unidentified archaeon SCA1150	0.574	0.001
FST	ASV62	Thaumarchaeota	Nitrososphaeria	Nitrososphaerales	Nitrososphaeraceae	Unidentified archaeon SCA1151	Unidentified archaeon SCA1151	0.819	0.001
FST	ASV63	Thaumarchaeota	Nitrososphaeria	Nitrososphaerales	Nitrososphaeraceae	Unidentified archaeon SCA1173	Unidentified archaeon SCA1173	0.181	0.011
FST	ASV65	Thaumarchaeota	Nitrososphaeria	Nitrosotaleales	Nitrosotaleaceae	Candidatus Nitrosotalea	Uncultured archaeon	0.451	0.001
FST	ASV69	Thaumarchaeota	Nitrososphaeria	Nitrosotaleales	Nitrosotaleaceae	NA	NA	0.241	0.001
FST	ASV66	Thaumarchaeota	Nitrososphaeria	Nitrosotaleales	Nitrosotaleaceae	Uncultured archaeon	Uncultured archaeon	0.685	0.001
FST	ASV67	Thaumarchaeota	Nitrososphaeria	Nitrosotaleales	Nitrosotaleaceae	Uncultured crenarchaeote	Uncultured crenarchaeote	0.246	0.001
FST	ASV71	Thaumarchaeota	SCGC AB-179-E04	Uncultured crenarchaeote	Uncultured crenarchaeote	Uncultured crenarchaeote	Uncultured crenarchaeote	0.154	0.035
WCC	ASV1	Crenarchaeota	Bathyarchaeia	Archaeon RBG_16_50_20	Archaeon RBG_16_50_20	Archaeon RBG_16_50_20	Archaeon RBG_16_50_20	0.332	0.001
WCC	ASV3189	Euryarchaeota	Methanobacteria	Methanobacteriales	Methanobacteriaceae	Methanobacterium	NA	0.327	0.001
WCC	ASV37	Nanoarchaeaeota	Woesearchaeia	Metagenome	Metagenome	Metagenome	Metagenome	0.549	0.001
WCC	ASV5035	Thaumarchaeota	Nitrososphaeria	Nitrososphaerales	Nitrososphaeraceae	Uncultured archaeon	Uncultured archaeon	0.269	0.001
CB	ASV3190	Euryarchaeota	Thermoplasmata	Marine Group II	Marine metagenome	Marine metagenome	Marine metagenome	0.209	0.001
CB	ASV15	Euryarchaeota	Thermoplasmata	Marine Group II	NA	NA	NA	0.172	0.001
CB	ASV3191	Euryarchaeota	Thermoplasmata	Marine Group II	Uncultured marine euryarchaeote DH148-W1	Uncultured marine euryarchaeote DH148-W1	Uncultured marine euryarchaeote DH148-W1	0.227	0.001
CB	ASV39	Nanoarchaeaeota	Woesearchaeia	Uncultured archaeon	Uncultured archaeon	Uncultured archaeon	Uncultured archaeon	0.214	0.002
CB	ASV40	Nanoarchaeaeota	Woesearchaeia	Uncultured bacterium	Uncultured bacterium	Uncultured bacterium	Uncultured bacterium	0.169	0.021
CB	ASV3197	Thaumarchaeota	Nitrososphaeria	Nitrosopumilales	Nitrosopumilaceae	Candidatus Nitrosopumilus	NA	0.377	0.001

*FST, agricultural soils; WCC, freshwater biofilms; CB, estuary.*

### Prediction of Archaeal Functional Profiles Across Habitats

**FIGURE 7:**
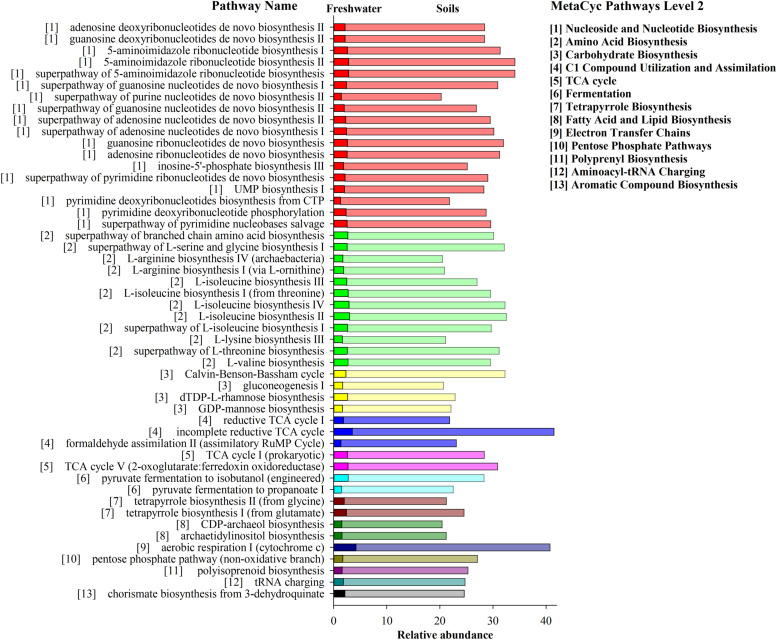
Bar graph showing the relative abundance of archaeal genomic signatures predicted by PICRUSt2 within MetaCyc categories for agricultural soils FST (dark bar) and freshwater biofilms WCC (light bar). The estuarine samples (CB) were omitted due to unidentified functions. Pathways are displayed on the y axis, the number (1–13) in front of pathway names correspond to the number (1–13) of MetaCyc Pathways Level 2 shown on the right.

The potential metabolic functions of archaeal communities and their proportions of occurrence in each step of metabolic processes were predicted by PICRUSt2. In total 352 functional pathways were found in this study. Significant differences of the contribution and quantity of the top 50 functional pathways in archaeal communities were observed across the three habitats (Figure 7). These pathways were classified to 13 main metabolic groups. The total percentage of these pathways ranged from 479.5 to 22.5% in soil samples and from 40.6 to 1.5% in freshwater biofilm samples, which included “Nucleoside and Nucleotide Biosynthesis,” “Amino Acid Biosynthesis,” “Carbohydrate Biosynthesis,” “C1 Compound Utilization and Assimilation,” “TCA cycle,” “Fermentation” and several other pathways (Figure 7). The predicted pathways were most abundant in soils, followed by freshwater biofilms, and were unidentified in estuary water samples. Those most abundant pathways in soils included “incomplete reductive TCA cycle,” “aerobic respiration I (cytochrome c),” “5-aminoimidazole ribonucleotide biosynthesis” and “Calvin-Benson-Bassham cycle” while the most abundant metabolism pathways in the freshwater biofilms were identified as “aerobic respiration I (cytochrome c),” “incomplete reductive TCA cycle,” “L-isoleucine biosynthesis II,” and “L-isoleucine biosynthesis IV” (Figure 7).

## Discussion

### Heterogeneous Distribution of Archaeal Groups Across Habitats

To gain knowledge on the true ecology of a Domain, all its components should be analyzed as a whole (Auguet et al., 2010). This comparative ecological study revealed detailed archaeal composition and community distribution across three habitats by high-throughput sequencing analysis. Distinct dominant archaeal groups and phylogenetic shifts were observed in three habitat-season combinations: agriculture soils from winter, freshwater biofilms from summer, and estuary waters from all seasons. Although Thaumarchaeota, Euryarchaeota, Nanoarchaeota, Crenarchaeota, and Diapherotrites were the most commonly found dominant groups ranging from soils to waters, they had uneven abundances among habitat types. Distinct archaeal abundances across different habitats or environments were also observed in earlier studies (Auguet et al., 2010; Wen et al., 2017; Alves et al., 2018). These variances in taxa abundances were present at both phylum and ASV levels (Figure 3 and Supplementary Table S3). Distinct archaeal taxa dominated different environments within each major phylum. For example, the proportion of Thaumarchaeota was significantly different across the three habitats (*P* < 0.01) (Figure 3), and clear phylogenetic shifts within the phylum were also observed (Supplementary Table S3). One member of Nitrosopumilaceae (ASV3197), probably a typical estuarine/brackish species, dominated the archaeal communities in the estuary surface water, but nearly unidentified in the other two habitats. Two other members of Nitrosopumilaceae (ASV54 and 64) were dominant in the freshwater biofilms, yet were nearly undetected in estuary waters. Furthermore, ASV66 (Nitrosotaleaceae) and ASV64 and 57 (Nitrosopumilaceae) dominated the soil environments (Supplementary Table S3). Thaumarchaeota are able to obtain ammonia from urea and cyanate (Baker et al., 2012; Palatinszky et al., 2015). Therefore, Thaumarchaeota have important links to climate change, as their activity has been linked to the production of the greenhouse gas nitrous oxide (N_2_O) through the oxidation of ammonia to nitrite and reduction of nitrite to N_2_O (Ostrom et al., 2000; Santoro et al., 2011). Thaumarchaeota are among the most abundant Archaea on the planet (Baker et al., 2020), including extreme environments such as the Yellowstone Lake and its lake floor hydrothermal vents (Kan et al., 2011). This study also demonstrates that Thaumarchaeota are relatively more abundant in soils than in the freshwater biofilms and estuary waters.

Phylogenetic shifts within a specific phylum were clearly observed among habitats (Supplementary Figure S2), such as those dominant taxa within Thaumarchaeota and Euryarchaeota (Supplementary Table S3 and Supplementary Figures S2C,E). Similar patterns were also found in global phylogeny and environmental distribution of ammonia-oxidizing archaea (Alves et al., 2018) and methanogenic archaea (Wen et al., 2017). Phylogenetic distribution of Crenarchaeota groups was mainly found in the soil environment and less in the freshwater and estuary water (Supplementary Figure S2A), while the most relatively abundant taxa in the Diapherotrites groups were retrieved from soil environment (Supplementary Figure S2B). Further, phylogenetic affiliation and shifts of Euryarchaeota, Nanoarchaeota and Thaumarchaeota were primarily identified in the soil and estuary water and less in freshwater biofilms (Supplementary Figures S2C–E). This implies that phylogenetically closely related archaeal organisms have adapted to very different habitats, and also reflects the broad environmental distribution of major archaeal groups such as Thaumarchaeota and Euryarchaeota (Auguet et al., 2010; Alves et al., 2018). Euryarchaeota contain the greatest number and diversity of cultured lineages (Baker et al., 2020). They are not just involved in methane production and anaerobic methane oxidation (Orphan et al., 2002), but also participate the anaerobic oxidation of other short-chain hydrocarbons (Wang et al., 2019), suggesting that these microbes have varied roles in biogeochemical cycles. Agricultural soils and freshwater biofilms also each had an indicator ASV from the Euryarchaeota that was capable of methane production. The phylogenetic parallels of specific taxa within each archaeal phylum that dominated different habitats provide opportunities to examine interesting evolutionary tracks between soils, freshwater biofilms, and estuary water lineages.

### Distinct Diversity and Distribution Patterns of Archaeal Communities

Differences in archaeal diversities and Bacteria:Archaea ratios were clearly shown among soils, freshwater biofilms, and estuary waters. The error bars to be large because the communities were sampled across time, seasons, biofilm succession, and different farming practices. Despite these factors corresponding with high variability of an Archaea community within a habitat over time and space, it is remarkable that the Archaea communities differ significantly among environments based on one-way ANOVA and ANOSIM analysis. We would be more surprised if there were very small error bars which could indicate lack of community changes over time and space (this would be highly unusual) or that the environments were not sufficiently sampled. Low Bacteria:Archaea ratios and most of the archaeal diversities were associated with soil environments, and as expected, they were different from freshwater biofilms and estuary (Figure 2). In fact, soil is the most diverse environment on Earth and hosts high bacterial and archaeal abundance and diversity (Griffiths et al., 2016). Almost 25% of the Bacteria and Archaea on Earth live in soils (to 8 m of depth), encompassing roughly 3 × 10^29^ cells, and those that live in the sea surface layer are only about 2 × 10^23^ (Flemming and Wuertz, 2019). Biofilms dominate all habitats on the surface of the Earth, except in the oceans, accounting for ∼80% of bacterial and archaeal cells. Biofilms drive the majority of biogeochemical processes and represent the main way of active bacterial and archaeal life (Flemming and Wuertz, 2019). Our study is consistent with previous studies and shows that archaeal communities are very abundant and diverse in soil compared to other environments on Earth (Baker et al., 2020), such as rivers and estuary waters.

Niche partitioning of archaeal communities clearly exists among soils, freshwater biofilms and estuarine surface waters (NMDS, Figure 4). Three different environments (soils, freshwater biofilms and estuary waters) differ with clear gradients in pH, oxygen and biologically relevant constituents such as CO_2_, CH_4_, and NH_4_, which likely contributed to niche separation and differentiation, leading to the structuring and distribution of distinct archaeal physiological types in different environments (Biller et al., 2012; Reichenberger et al., 2015; Alves et al., 2018). Therefore, strong geochemical signatures (Supplementary Table S1) across three environments provide numerous niches capable of supporting phylogenetically and functionally diverse archaeal populations. For example, certain groups of Archaea that preferentially inhabit temperate estuarine surface waters such as ASV3197 (Nitrosopumilaceae) and two members of Marine Group II (ASV15 and ASV3190) (Supplementary Table S3). Similar patterns were also observed in earlier studies indicating that some microbial groups prefer to inhabit estuaries (Caporaso et al., 2011; Wen et al., 2017), even with strong geographical differentiation of archaeal communities across global estuaries (Liu et al., 2018). One important factor to recognize from this study is that our conclusions are intertwined with the season or seasons in which they were sampled. Seasonal categories can have a major influence on microbial community composition within an environment (Wang et al., 2020). For example, Hullar et al. (2005) identified phototrophic-driven seasonal shifts in epilithic bacteria communities collected at the same White Clay Creek site (Pennsylvania Piedmont) used for this study. However, we have observed that for bacteria, season explains less of the variability in community composition than different environments within White Clay Creek (unpublished data). Because Bacteria and Archaea community samples cluster together in the NMDS, we surmise that for this study, seasonal differences within freshwater biofilms are unlikely to be more influential than those between freshwater biofilms and the other environments. While seasonal changes also occur in agricultural soils (e.g., Bossio et al., 1998 although they identified soil type as more influential than time), our DNA-based approach may have integrated soil archaeal composition across seasons. Carini et al. (2020) have shown that removal of relic DNA from a soil enhances detection of prokaryote community temporal patterns, suggesting that some composition of the community is retained from season-to-season. Therefore, in the future, there is a great need for detailed investigations and comparisons of how the archaeal community structure responds to seasonal as well as spatial variations across different environments. However, according to the nature of habitat and environment, it is necessary to carefully consider measurement of environmental parameters and the sampling strategies covering both time and space.

Our study also reveals that the relative abundances of archaeal communities from different habitats have extremely uneven phylogenetic diversities, with few clades overwhelmingly dominating overall archaeal diversity in a specific environment. For example, most members of the Thaumarchaeota (21 out of 22) were affiliated with the class Nitrososphaeria, including those taxa abundant across the three habitats, such as ASV57, ASV64, ASV66, and ASV3197 (Supplementary Table S3). Ammonia-oxidizing Archaea (AOA) comprise a diverse group of organisms formally defined as class Nitrososphaeria of the phylum Thaumarchaeota (Rosenberg et al., 2014; Baker et al., 2020). Plenty of amoA-based studies collectively have shown that AOA diversity and abundance in nature depend on multiple factors and are strongly partitioned by local environments, and that AOA plays a major role in nitrification, the conversion of ammonia to nitrate via nitrite (Francis et al., 2005; Biller et al., 2012; Stahl and de la Torre, 2012; Alves et al., 2018). Different ecosystems tend to harbor distinct AOA groups and niche adaptation directly or indirectly contributes to the selection of specific archaeal groups. Our study shows that considerable habitat specificity and Archaeal diversification reflects diverse niche adaptation. This potentially implies that AOA are ubiquitous and abundant from soils to freshwaters to estuaries but have uneven distribution patterns.

### Differential Archaeal Networks, Indicator Species, and Functions

Co-occurrence network analysis indicated that only soil Archaea formed complex networks (29 taxa with 68 correlations) and key species (ASVs) were identified mainly from Methanomassiliicoccales, Nitrososphaerales, Nitrosopumilales (Figure 5). In addition, these key species were also identified as indicator species in the soil environment, suggesting unique adaptation to, or preference for, soil environments by taxa within these groups. Compared to soils, less archaeal diversity and abundance occurred in freshwater biofilms and surface water in estuaries. We speculate these archaea, either attached or free-living ones, may be more dependent on interaction with other biomes such as prokaryotes (i.e., Bacteria) or eukaryotes (e.g., microalgae). Though these biotic interactions have not been well documented and characterized, previous observations have shown that occurrence and abundances of archaeal groups coincide with diatoms, cyanobacteria, and viruses (Lima-Mendez et al., 2015; Needham and Fuhrman, 2016; Xie et al., 2018). Moreover, the potential of Archaea to shape their surroundings by a profound interaction with their biotic and abiotic environment has been researched (Valentine, 2007; Morris et al., 2013; Comolli and Banfield, 2014; Wegener et al., 2015). Moissl-Eichinger et al. (2018) summarized the basic principles of archaeal interactions, which are mainly based on the following driving factors: energetic pressure deriving from the environment, the capability for exchange of metabolites and/or electrons, genomic and structural adaptation capacity (by symbiont and host), and detoxification or facilitated horizontal gene transfer.

To investigate the potential biogeochemical implications of archaeal ASVs switching across environments, the functional capacity of the soil-, freshwater biofilm-, and estuary water-associated archaeal communities was analyzed using existing genomes within PICRUSt2 (Langille et al., 2013; Douglas et al., 2019). The predictions are sparse or lacking when PICRUSt 2 is applied using phylogenetic marker gene signatures from lesser known environments, such as the estuary Chesapeake Bay. This might be the main reason why archaeal functions were unidentified in those archaeal communities in the Bay. Although this technique is limited by the ability of 16S rRNA gene sequences to resolve ecologically important units and the phylogenetic breadth and depth of archaeal genomes, metagenomics prediction may nevertheless offer insight into the extent of both functional redundancy and differences in biogeochemical potential (but not rates) across natural environments.

Predicted pathways from the environments investigated here were most abundant in soils indicating that archaeal communities had much higher metabolic activities in the soil environments compared to the freshwater biofilms and estuary water. These predicted functional profiles are consistent with the proportion of major archaeal groups across the three environments, such as Thaumarchaeota and Nanoarchaeota which are more relatively abundant in soils than the other two environments. Earlier studies also showed distinct metabolic features of microbial communities across different environments, including water, mineral fractions, and microbial biofilms (Mesa et al., 2017). Archaeal communities in soils contain stronger abilities to perform Biosynthesis (e.g., Nucleoside and Nucleotide, Amino Acid, carbohydrate, Fatty acid, and Lipid), Generation of Precursor Metabolites and Energy (e.g., TCA cycle, Fermentation, and Electron Transfer Chains), and Degradation/Utilization/Assimilation (C1 Compound Utilization and Assimilation). The most abundant pathway in the soil samples, “incomplete reductive TCA cycle,” was performed exclusively by and widely observed in the archaeal groups of Methanobacteria, Methanococci, Methanomicrobia, including the following taxa: *Methanococcus maripaludis*, *Methanospirillum hungatei*, *Methanothermobacter thermautotrophicus*. Similarly, the pathways of “inosine-5′-phosphate biosynthesis III,” “archaetidylinositol biosynthesis,” “CDP-archaeol biosynthesis,” and “phosphopantothenate biosynthesis III (archaebacteria)” were performed exclusively by Archaea, including Methanocaldococcus, Methanothermobacter, *Archaeoglobus fulgidus*, *Methanosarcina acetivorans*, *Thermococcus kodakarensis*. In addition, “aerobic respiration I (cytochrome c),” “incomplete reductive TCA cycle,” “L-isoleucine biosynthesis II,” and “L-isoleucine biosynthesis” were the most abundant metabolism pathways in freshwater biofilms, and they were mostly found in the archaeal groups of Methanobacteria, Methanococci, Methanomicrobia, and Thermoprotei. Microbiome functions were found to be responsible for interactions via nutrient exchange, but also for coping with environmental stress, to which Archaea are in general evolutionarily adapted (Valentine, 2007; Moissl-Eichinger et al., 2018). Overall, habitat differentiation from soil to freshwater to estuary could alter greatly the biogeochemical potential of archaeal communities with apparent replacement by distinct archaeal groups under different environments.

In general, archaeal networks, indicator species and their functions under each habitat further confirmed that environmental selection/adaptation has a great effect in shaping archaeal communities (Offre et al., 2013; Baker et al., 2020) and provides further evidence and knowledge on the biodiversity and complexity of archaeal communities across environmental ecosystems. Future efforts could focus on quantitative assessments of targeted archaeal groups (e.g., ammonia-oxidizing Archaea or methanogenic Archaea) and how they respond to their ambient environmental gradients in order to more precisely estimate their abundance, population dynamics and functional roles across environments.

## Conclusion

We analyzed and compared the structure, distribution, diversity, network, indicator species, and potential functions of archaeal communities among agriculture soils, freshwater biofilms, and estuarine surface waters with 16S amplicon high-throughput sequencing. Our study highlights the heterogeneous proportions of archaeal phyla and taxa from soils to estuary, and reflects the significant influence of environment dissimilarities on archaeal abundance. Differential distribution patterns and diversity of archaeal communities in specific environments suggest potential niche-specific features of Archaea from soil, freshwater biofilms, and estuaries. Archaeal communities have complex networks, high metabolic activities and different indicator species in soil environments compared to freshwater biofilms and estuarine waters. The pressure of niche adaptation can contribute greatly to the variation of Archaea across the three habitats. This study shows the strong differentiation of archaeal communities from distinct ecosystems and provides guidance for the discovery of global diversity, distribution pattern, and ecological significance of Archaea.

## Data Availability Statement

The datasets presented in this study can be found in online repositories. The names of the repository/repositories and accession number(s) can be found in the article/Supplementary Material.

## Author Contributions

JK, FC, and CZ designed this research project. HW, RB, and LZ analyzed the data. RB, MP, EO, AM, and JK performed the experiments and collected the samples. HW, RB, MP, EO, AM, FC, CZ, and JK contributed to manuscript draft, tables, and figures, and approved the submitted version. All authors contributed to the article and approved the submitted version.

## Conflict of Interest

The authors declare that the research was conducted in the absence of any commercial or financial relationships that could be construed as a potential conflict of interest.

## Footnotes

1 https://support.illumina.com/

2 https://github.com/qiime2/q2-feature-classifier

3 https://github.com/ryanjw/co-occurrence

4 https://github.com/picrust/picrust2/wiki/q2-picrust2-Tutorial
