# Supplementary material for: Distinct Distribution of Archaea From Soil to Freshwater to Estuary: Implications of Archaeal Composition and Function in Different Environments
Source: Front Microbiol. 2020 Oct 22;11:576661. doi: 10.3389/fmicb.2020.576661 (PMC7642518; doi:10.3389/fmicb.2020.576661)
Supplement: Supplementary file 1 [file Presentation_1.pdf]

Supplementary Materials

Distinct distribution of Archaea from soil to freshwater to estuary: implications of archaeal composition and function in different environments

There are 3 tables and 3 figures in the supplemental information.

Supplementary Table S1. Physico-chemical parameters across the three environments: agricultural soils, freshwater, and estuary surface waters.

Supplementary Table S2. Statistics of quality control filtering of 16S rRNA gene sequences and the percentage of clean 16S rRNA gene sequences dominated by Archaea in our samples. FST = agricultural soils, WCC = freshwater biofilms, CB = estuary.

Supplementary Table S3. Major archaeal taxa across the three habitats. ASVs were filtered out if they occurred in less than five samples and had a summed relative abundance of less than 0.1%.

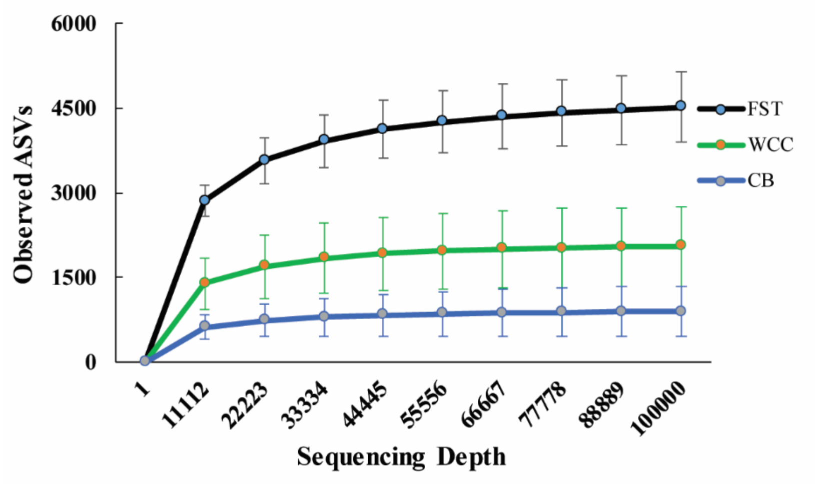

Supplementary Figure S1. Rarefaction curves to show the effects of sequencing efforts on the observed number of ASVs in our samples. Curves were calculated to the normalized subsample size of 100,000 sequences in the three environments (FST = agricultural soil, WCC = freshwater biofilm and CB = estuary). Bars represent standard error of the mean of the samples from each environment.

A

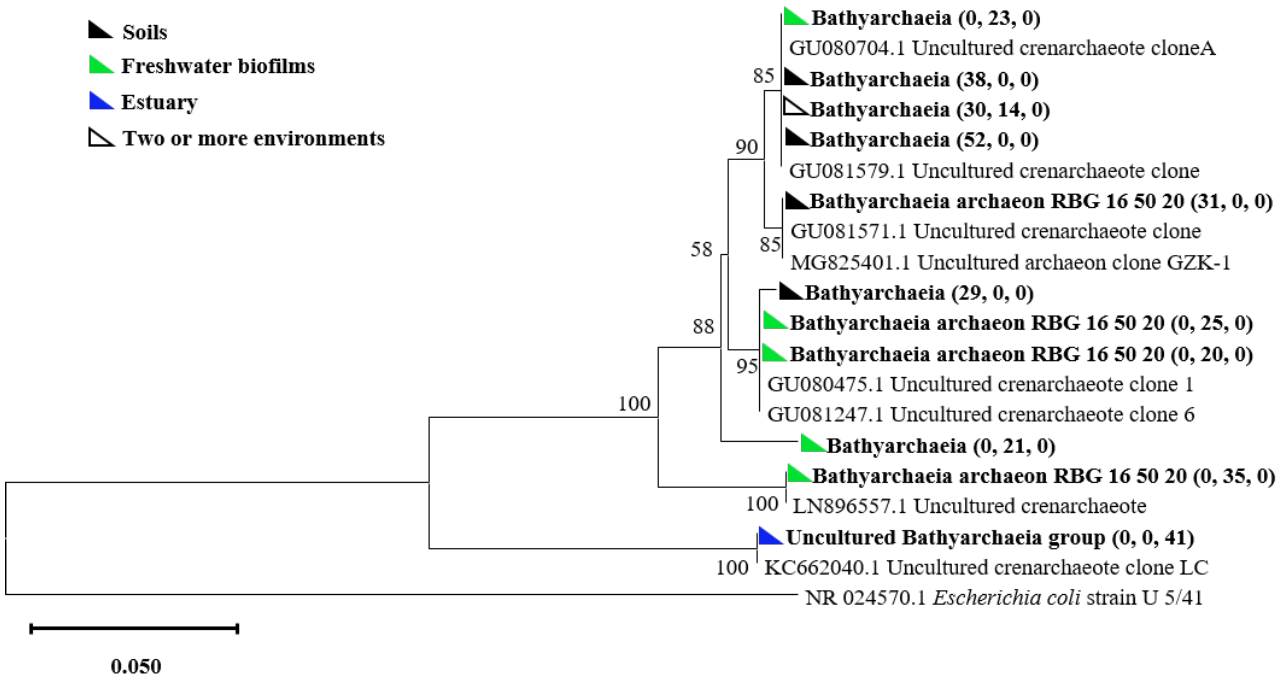

Soils
Freshwater biofilms
Estuary
Two or more environments
Bathyarchaeia (0, 23, 0)
GU080704.1 Uncultured crenarchaeote cloneA
Bathyarchaeia (38, 0, 0)
Bathyarchaeia (30, 14, 0)
Bathyarchaeia (52, 0, 0)
GU081579.1 Uncultured crenarchaeote clone
Bathyarchaeia archaeon RBG 16 50 20 (31, 0, 0)
GU081571.1 Uncultured crenarchaeote clone
MG825401.1 Uncultured archaeon clone GZK-1
Bathyarchaeia (29, 0, 0)
Bathyarchaeia archaeon RBG 16 50 20 (0, 25, 0)
Bathyarchaeia archaeon RBG 16 50 20 (0, 20, 0)
GU080475.1 Uncultured crenarchaeote clone 1
GU081247.1 Uncultured crenarchaeote clone 6
Bathyarchaeia (0, 21, 0)
Bathyarchaeia archaeon RBG 16 50 20 (0, 35, 0)
LN896557.1 Uncultured crenarchaeote
Uncultured Bathyarchaeia group (0, 0, 41)
KC662040.1 Uncultured crenarchaeote clone LC
NR 024570.1 Escherichia coli strain U 5/41
0.050

B

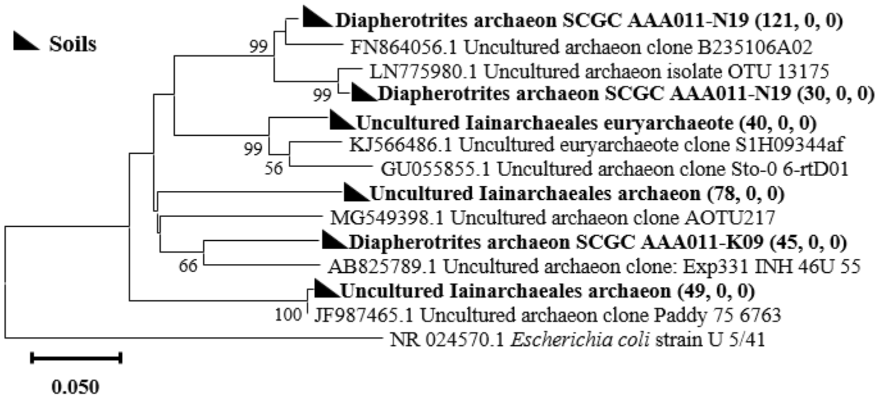

Soils
Diapherotrites archaeon SCGC AAA011-N19 (121, 0, 0)
FN864056.1 Uncultured archaeon clone B235106A02
LN775980.1 Uncultured archaeon isolate OTU 13175
Diapherotrites archaeon SCGC AAA011-N19 (30, 0, 0)
Uncultured Iainarchaeales euryarchaeote (40, 0, 0)
KJ566486.1 Uncultured euryarchaeote clone S1H09344af
GU055855.1 Uncultured archaeon clone Sto-0 6-rtD01
Uncultured Iainarchaeales archaeon (78, 0, 0)
MG549398.1 Uncultured archaeon clone AOTU217
Diapherotrites archaeon SCGC AAA011-K09 (45, 0, 0)
AB825789.1 Uncultured archaeon clone: Exp331 INH 46U 55
Uncultured Iainarchaeales archaeon (49, 0, 0)
JF987465.1 Uncultured archaeon clone Paddy 75 6763
NR 024570.1 Escherichia coli strain U 5/41
0.050

C

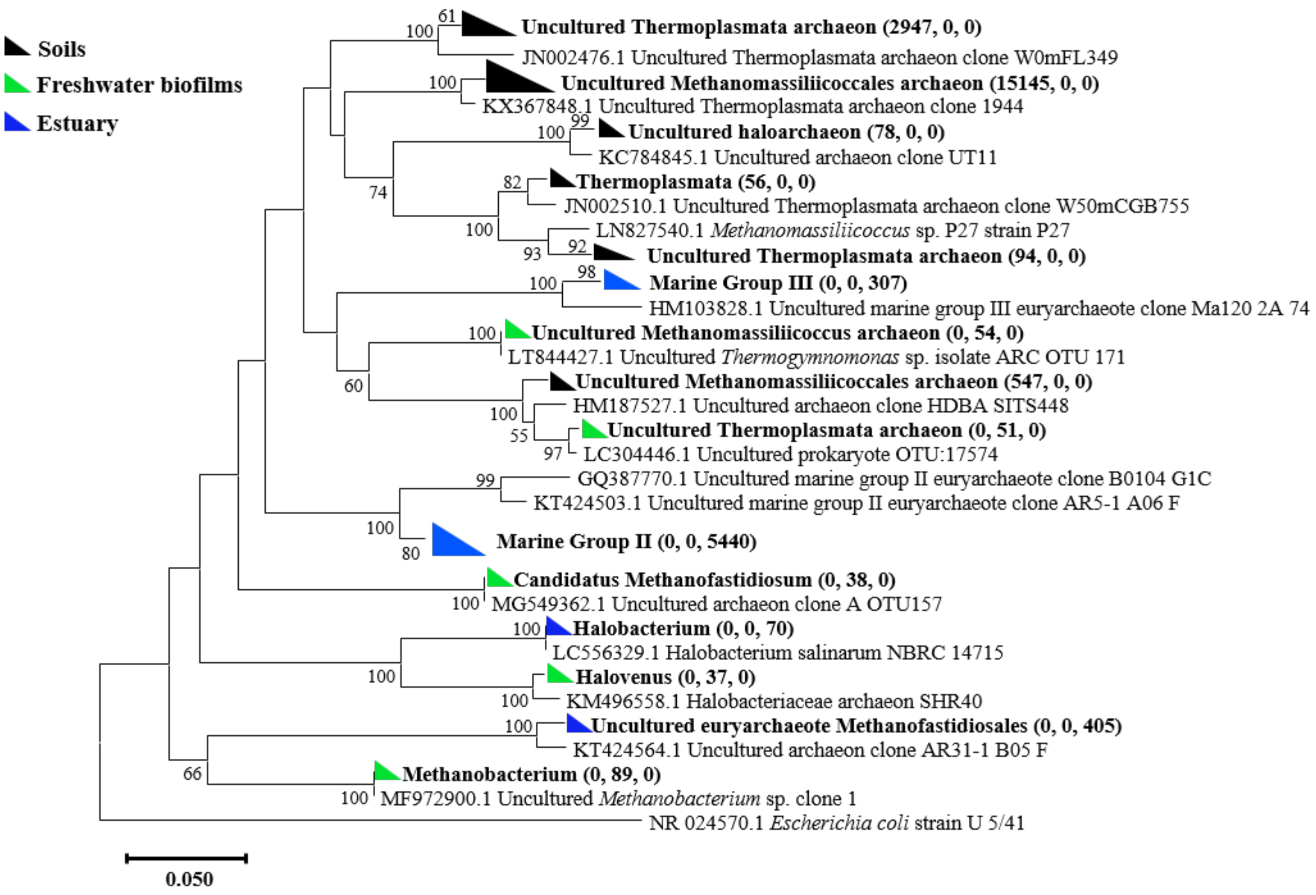
Soils
Freshwater biofilms
Estuary
Uncultured Thermoplasmata archaeon (2947, 0, 0)
JN002476.1 Uncultured Thermoplasmata archaeon clone W0mFL349
Uncultured Methanomassiliicoccales archaeon (15145, 0, 0)
KX367848.1 Uncultured Thermoplasmata archaeon clone 1944
Uncultured haloarchaeon (78, 0, 0)
KC784845.1 Uncultured archaeon clone UT11
Thermoplasmata (56, 0, 0)
JN002510.1 Uncultured Thermoplasmata archaeon clone W50mCGB755
LN827540.1 Methanomassiliicoccus sp. P27 strain P27
Uncultured Thermoplasmata archaeon (94, 0, 0)
Marine Group III (0, 0, 307)
HM103828.1 Uncultured marine group III euryarchaeote clone Ma120 2A 74
Uncultured Methanomassiliicoccus archaeon (0, 54, 0)
LT844427.1 Uncultured Thermogymnomonas sp. isolate ARC OTU 171
Uncultured Methanomassiliicoccales archaeon (547, 0, 0)
HM187527.1 Uncultured archaeon clone HDBA SITS448
Uncultured Thermoplasmata archaeon (0, 51, 0)
LC304446.1 Uncultured prokaryote OTU:17574
GQ387770.1 Uncultured marine group II euryarchaeote clone B0104 G1C
KT424503.1 Uncultured marine group II euryarchaeote clone AR5-1 A06 F
Marine Group II (0, 0, 5440)
Candidatus Methanofastidiosum (0, 38, 0)
MG549362.1 Uncultured archaeon clone A OTU157
Halobacterium (0, 0, 70)
LC556329.1 Halobacterium salinarum NBRC 14715
Halovenus (0, 37, 0)
KM496558.1 Halobacteriaceae archaeon SHR40
Uncultured euryarchaeote Methanofastidiosales (0, 0, 405)
KT424564.1 Uncultured archaeon clone AR31-1 B05 F
Methanobacterium (0, 89, 0)
MF972900.1 Uncultured Methanobacterium sp. clone 1
NR 024570.1 Escherichia coli strain U 5/41
0.050

D

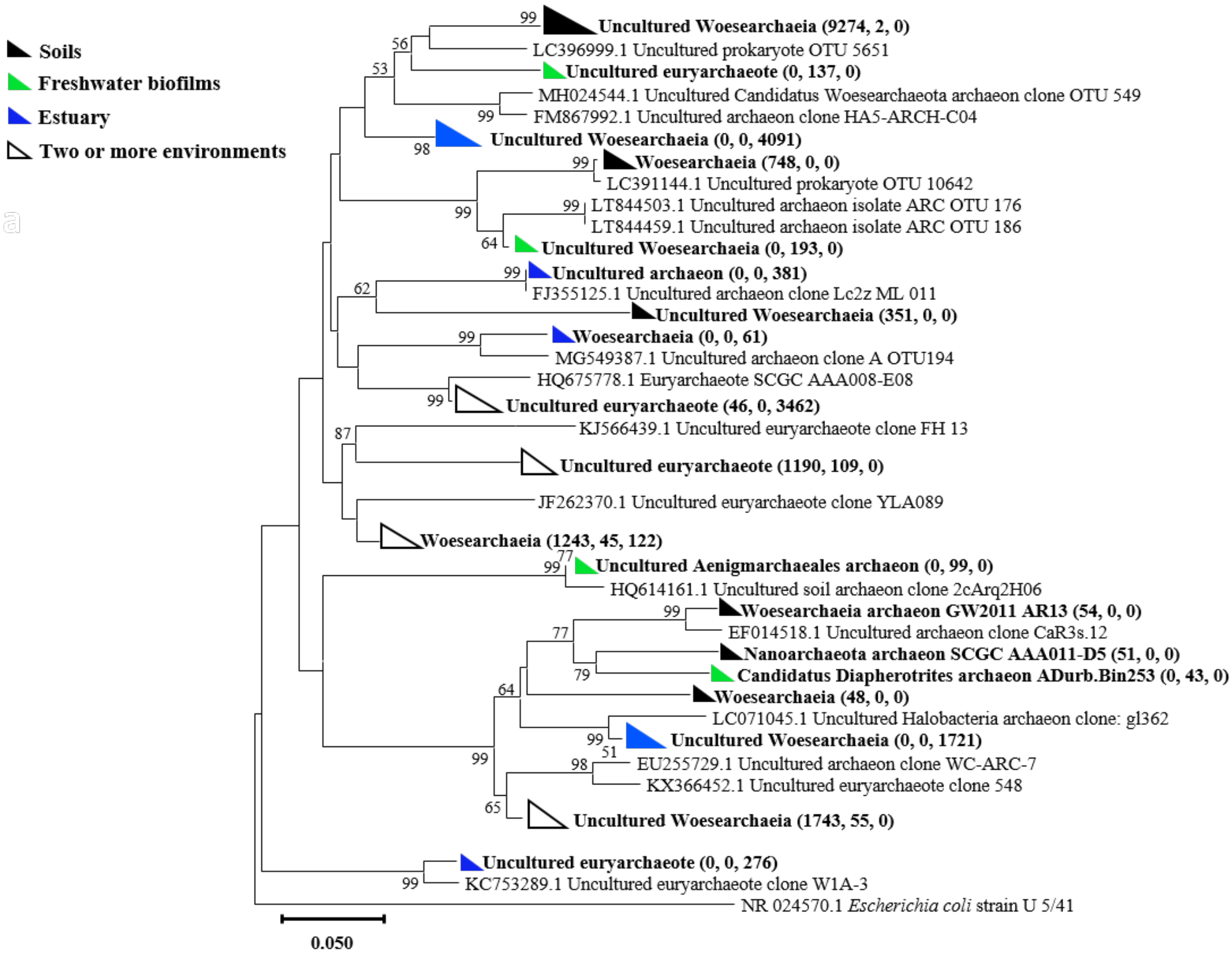
Soils
Freshwater biofilms
Estuary
Two or more environments
Uncultured Woesearchaeia (9274, 2, 0)
LC396999.1 Uncultured prokaryote OTU 5651
Uncultured euryarchaeote (0, 137, 0)
MH024544.1 Uncultured Candidatus Woesearchaeota archaeon clone OTU 549
FM867992.1 Uncultured archaeon clone HA5-ARCH-C04
Uncultured Woesearchaeia (0, 0, 4091)
Woesearchaeia (748, 0, 0)
LC391144.1 Uncultured prokaryote OTU 10642
LT844503.1 Uncultured archaeon isolate ARC OTU 176
LT844459.1 Uncultured archaeon isolate ARC OTU 186
Uncultured Woesearchaeia (0, 193, 0)
Uncultured archaeon (0, 0, 381)
FJ355125.1 Uncultured archaeon clone Lc2z ML 011
Uncultured Woesearchaeia (351, 0, 0)
Woesearchaeia (0, 0, 61)
MG549387.1 Uncultured archaeon clone A OTU194
HQ675778.1 Euryarchaeote SCGC AAA008-E08
Uncultured euryarchaeote (46, 0, 3462)
KJ566439.1 Uncultured euryarchaeote clone FH 13
Uncultured euryarchaeote (1190, 109, 0)
JF262370.1 Uncultured euryarchaeote clone YLA089
Woesearchaeia (1243, 45, 122)
Uncultured Aenigmarchaeales archaeon (0, 99, 0)
HQ614161.1 Uncultured soil archaeon clone 2cArq2H06
Woesearchaeia archaeon GW2011 AR13 (54, 0, 0)
EF014518.1 Uncultured archaeon clone CaR3s.12
Nanoarchaeota archaeon SCGC AAA011-D5 (51, 0, 0)
Candidatus Diapherotrites archaeon ADurb.Bin253 (0, 43, 0)
Woesearchaeia (48, 0, 0)
LC071045.1 Uncultured Halobacteria archaeon clone: gl362
Uncultured Woesearchaeia (0, 0, 1721)
EU255729.1 Uncultured archaeon clone WC-ARC-7
KX366452.1 Uncultured euryarchaeote clone 548
Uncultured Woesearchaeia (1743, 55, 0)
Uncultured euryarchaeote (0, 0, 276)
KC753289.1 Uncultured euryarchaeote clone W1A-3
NR 024570.1 Escherichia coli strain U 5/41
0.050

E

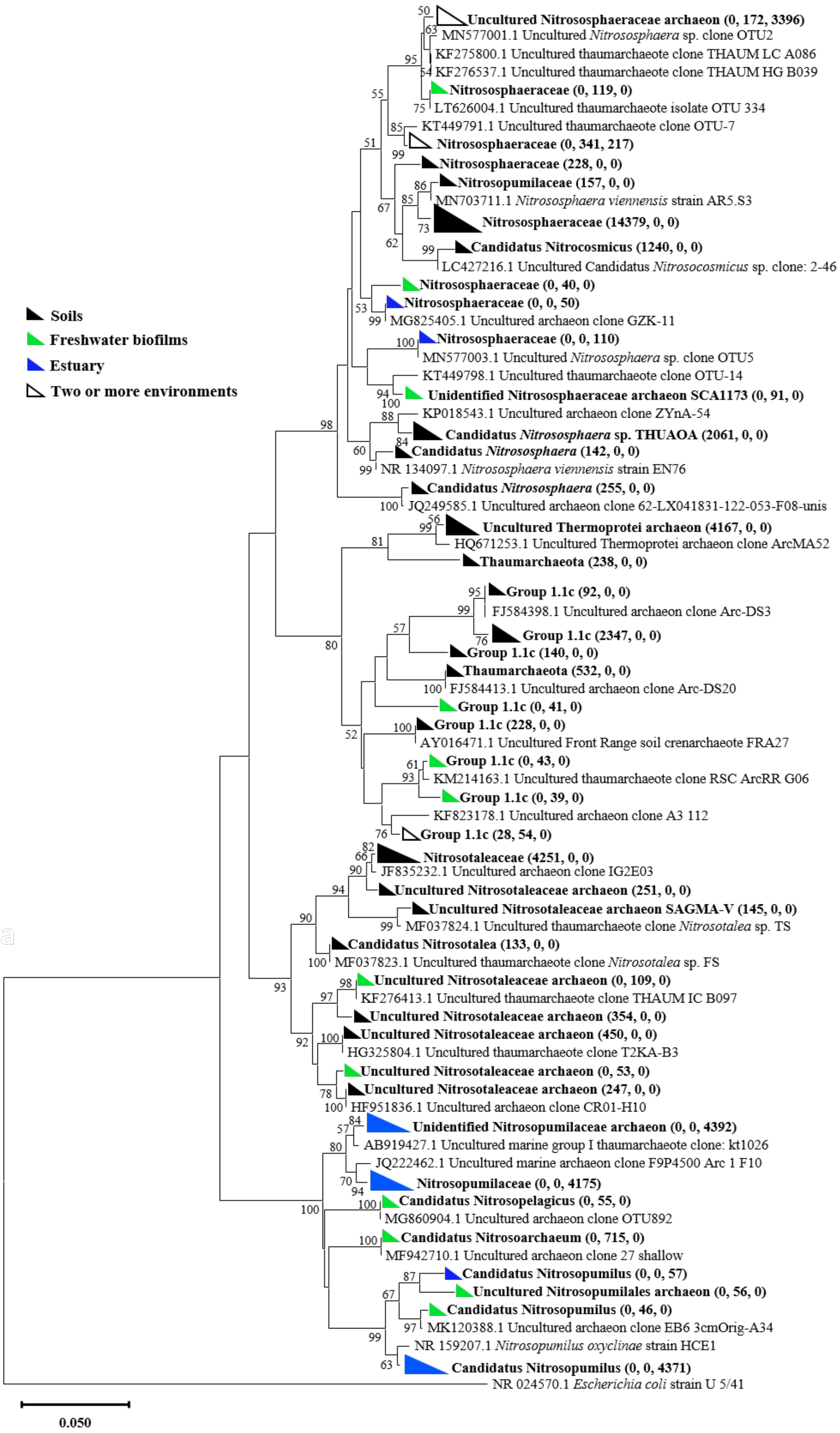
Uncultured Nitrososphaeraceae archaeon (0, 172, 3396)
MN577001.1 Uncultured Nitrososphaera sp. clone OTU2
KF275800.1 Uncultured thaumarchaeote clone THAUM LC A086
KF276537.1 Uncultured thaumarchaeote clone THAUM HG B039
Nitrososphaeraceae (0, 119, 0)
LT626004.1 Uncultured thaumarchaeote isolate OTU 334
KT449791.1 Uncultured thaumarchaeote clone OTU-7
Nitrososphaeraceae (0, 341, 217)
Nitrososphaeraceae (228, 0, 0)
Nitrosopumilaceae (157, 0, 0)
MN703711.1 Nitrososphaera viennensis strain AR5.S3
Nitrososphaeraceae (14379, 0, 0)
Candidatus Nitrocosmicus (1240, 0, 0)
LC427216.1 Uncultured Candidatus Nitrosocosmicus sp. clone: 2-46
Nitrososphaeraceae (0, 40, 0)
Nitrososphaeraceae (0, 0, 50)
MG825405.1 Uncultured archaeon clone GZK-11
Nitrososphaeraceae (0, 0, 110)
MN577003.1 Uncultured Nitrososphaera sp. clone OTU5
KT449798.1 Uncultured thaumarchaeote clone OTU-14
Unidentified Nitrososphaeraceae archaeon SCA1173 (0, 91, 0)
KP018543.1 Uncultured archaeon clone ZYnA-54
Candidatus Nitrososphaera sp. THUAOA (2061, 0, 0)
Candidatus Nitrososphaera (142, 0, 0)
NR 134097.1 Nitrososphaera viennensis strain EN76
Candidatus Nitrososphaera (255, 0, 0)
JQ249585.1 Uncultured archaeon clone 62-LX041831-122-053-F08-unis
Uncultured Thermoprotei archaeon (4167, 0, 0)
HQ671253.1 Uncultured Thermoprotei archaeon clone ArcMA52
Thaumarchaeota (238, 0, 0)
Group 1.1c (92, 0, 0)
FJ584398.1 Uncultured archaeon clone Arc-DS3
Group 1.1c (2347, 0, 0)
Group 1.1c (140, 0, 0)
Thaumarchaeota (532, 0, 0)
FJ584413.1 Uncultured archaeon clone Arc-DS20
Group 1.1c (0, 41, 0)
Group 1.1c (228, 0, 0)
AY016471.1 Uncultured Front Range soil crenarchaeote FRA27
Group 1.1c (0, 43, 0)
KM214163.1 Uncultured thaumarchaeote clone RSC ArcRR G06
Group 1.1c (0, 39, 0)
KF823178.1 Uncultured archaeon clone A3 112
Group 1.1c (28, 54, 0)
Nitrosotaleaceae (4251, 0, 0)
JF835232.1 Uncultured archaeon clone IG2E03
Uncultured Nitrosotaleaceae archaeon (251, 0, 0)
Uncultured Nitrosotaleaceae archaeon SAGMA-V (145, 0, 0)
MF037824.1 Uncultured thaumarchaeote clone Nitrosotalea sp. TS
Candidatus Nitrosotalea (133, 0, 0)
MF037823.1 Uncultured thaumarchaeote clone Nitrosotalea sp. FS
Uncultured Nitrosotaleaceae archaeon (0, 109, 0)
KF276413.1 Uncultured thaumarchaeote clone THAUM IC B097
Uncultured Nitrosotaleaceae archaeon (354, 0, 0)
Uncultured Nitrosotaleaceae archaeon (450, 0, 0)
HG325804.1 Uncultured thaumarchaeote clone T2KA-B3
Uncultured Nitrosotaleaceae archaeon (0, 53, 0)
Uncultured Nitrosotaleaceae archaeon (247, 0, 0)
HF951836.1 Uncultured archaeon clone CR01-H10
Unidentified Nitrosopumilaceae archaeon (0, 0, 4392)
AB919427.1 Uncultured marine group I thaumarchaeote clone: kt1026
JQ222462.1 Uncultured marine archaeon clone F9P4500 Arc 1 F10
Nitrosopumilaceae (0, 0, 4175)
Candidatus Nitrosopelagicus (0, 55, 0)
MG860904.1 Uncultured archaeon clone OTU892
Candidatus Nitrosoarchaeum (0, 715, 0)
MF942710.1 Uncultured archaeon clone 27 shallow
Candidatus Nitrosopumilus (0, 0, 57)
Uncultured Nitrosopumilales archaeon (0, 56, 0)
Candidatus Nitrosopumilus (0, 46, 0)
MK120388.1 Uncultured archaeon clone EB6 3cmOrig-A34
NR 159207.1 Nitrosopumilus oxyclinae strain HCE1
Candidatus Nitrosopumilus (0, 0, 4371)
NR 024570.1 Escherichia coli strain U 5/41
Soils
Freshwater biofilms
Estuary
Two or more environments
0.050

Supplementary Figure S2. Phylogenetic trees constructed to show the most abundant reads (counts great than 20 across all samples) from the major archaeal phla including Crenarchaeota (A), Diapherotrites (B), Euryarchaeota (C), Nanoarchaeota (D), Thaumarchaeota (E). Sequences from the current study are highlighted in bold in the trees. In turn, the three numbers in parentheses indicated the counts of ASVs retrieved from soils, freshwater biofilms, and estuary waters. Unique sequence clusters from each sampling environments were shown with color-filled triangles: black (soils), green (freshwater biofilms), and blue (estuary). Open triangles were used to represent the sequences occurred from two or more sampling environments. The tree was built using neighbor-joining calculations in MEGA-X with 253 positions and 1000 resamplings. Bootstrap values >50% were displayed.

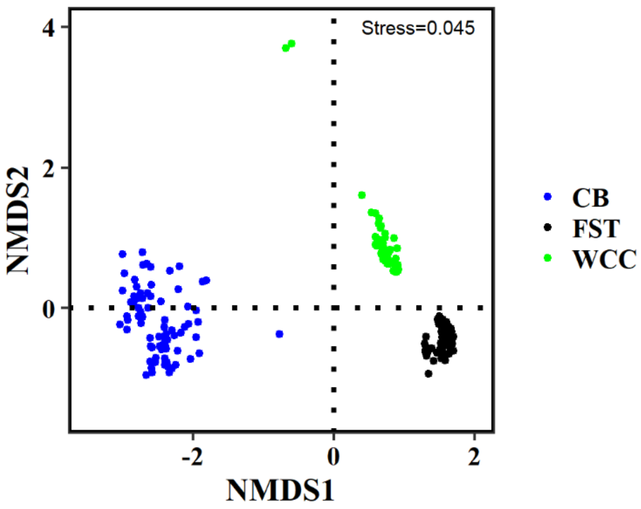

Supplementary Figure S3. Non-metric multidimensional scaling (NMDS) plots of Bacteria and Archaea communities across three environments. FST = agricultural soils, WCC = freshwater biofilms, CB = estuary.
